# Vitreous advanced glycation endproducts and α-dicarbonyls in retinal detachment patients with type 2 diabetes mellitus and non-diabetic controls

**DOI:** 10.1371/journal.pone.0173379

**Published:** 2017-03-06

**Authors:** Bernardina T. Fokkens, Douwe J. Mulder, Casper G. Schalkwijk, Jean L. Scheijen, Andries J. Smit, Leonoor I. Los

**Affiliations:** 1 Department of Internal Medicine, University of Groningen, University Medical Center Groningen (UMCG), Groningen, the Netherlands; 2 Research Institute GUIDE, Graduate School of Medical Sciences, University of Groningen, University Medical Center Groningen (UMCG), Groningen, the Netherlands; 3 Department of Ophthalmology, University of Groningen, University Medical Center Groningen (UMCG), the Netherlands; 4 Laboratory for Metabolism and Vascular Medicine, Maastricht University, Department of Experimental Internal Medicine, Maastricht, the Netherlands; 5 W.J. Kolff Institute, Graduate School of Medical Sciences, University of Groningen, University Medical Center Groningen (UMCG), Groningen, the Netherlands; Medizinische Universitat Graz, AUSTRIA

## Abstract

**Purpose:**

Advanced glycation endproducts (AGEs) and their precursors α-dicarbonyls are implicated in the progression of diabetic retinopathy. The purpose of this study was to assess AGEs and α-dicarbonyls in the vitreous of patients with type 2 diabetes mellitus (T2DM) with early stages or absence of diabetic retinopathy.

**Methods:**

We examined vitreous samples obtained during vitrectomy from 31 T2DM patients presenting themselves with rhegmatogenous retinal detachment and compared these to 62 non-diabetic rhegmatogenous retinal detachment patients, matched on age, estimated glomerular filtration rate, smoking, intra-ocular lens implantation, and proliferative vitreoretinopathy. AGEs (pentosidine, N^ε^-(carboxymethyl)lysine, N^ε^-(carboxyethyl)lysine, and 5-hydro-5-methylimidazolone) and α-dicarbonyls (3-deoxyglucosone, methylglyoxal, and glyoxal) were measured by ultra performance liquid chromatography or high performance liquid chromatography. Skin autofluorescence was measured by the AGE Reader.

**Results:**

Mean age was 64 ± 7.6 years for T2DM patients and 63 ± 8.1 years for controls. For T2DM patients, median diabetes duration was 2.2 (0.3–7.4) years. Non-proliferative diabetic retinopathy was present in 1 patient and classified as absent or background retinopathy in 30 patients. Vitreous levels of pentosidine (2.20 vs. 1.59 μmol/mol lysine, p = 0.012) and 3-deoxyglucosone (809 vs. 615 nmol/L, p = 0.001) were significantly elevated in T2DM patients compared to controls. Other AGEs and α-dicarbonyls in the vitreous were not significantly different. There was a trend for increased skin autofluorescence in T2DM patients as compared to controls (p = 0.07).

**Conclusions:**

Pentosidine and 3-deoxyglucosone concentrations were increased in the vitreous of rhegmatogenous retinal detachment patients with a relatively short duration of diabetes compared to non-diabetic rhegmatogenous retinal detachment patients.

## Introduction

Advanced glycation endproducts (AGEs) are thought to be involved in the pathogenesis of many age-related diseases, such as diabetes mellitus, atherosclerosis, cataract and Alzheimer’s disease. AGEs are formed by glycation and oxidation of free amino groups of proteins, lipids and nucleic acids. This metabolic process is complex and heterogeneous, yielding numerous different AGE adducts, such as pentosidine, N^ε^-(carboxymethyl)lysine (CML), N^ε^-(carboxyethyl)lysine (CEL) and 5-hydro-5-methylimidazolone (MG-H1)[1].

In diabetes, prolonged hyperglycemia and oxidative stress accelerate the accumulation of some AGEs. Besides via the classical Maillard reaction, AGEs are formed through the reaction of α-dicarbonyls, such as 3-deoxyglucosone (3-DG), methylglyoxal (MGO), and glyoxal (GO), with protein amino groups[2]. Accumulation of AGEs also strongly depends on tissue turnover because AGEs are mainly irreversibly linked to tissue proteins. Therefore, tissues with slow turnover (such as the skin, lens, and cartilage) capture decades-long glycaemia. In tissues with fast turnover (such as plasma, epidermis and mucosa), AGEs accumulate to a lesser extent since they are rapidly broken down to AGE peptides or free AGEs, which are excreted through the kidney[3].

AGEs promote tissue dysfunction *in vitro* and *in vivo* by altering protein structure, by cross-linking of long lived molecules and through binding to the receptor for advanced glycation endproducts (RAGE)[1,4]. Both α-dicarbonyls and AGEs have been linked to diabetic complications and have been postulated to play a pathological role in the development of these complications[5–7].

Several studies have shown elevated serum and vitreous AGE levels in diabetic retinopathy (DR)[8,9]. Furthermore, AGEs have been suggested to influence the transition of retinopathy from the non-proliferative to the proliferative state[10]. However, vitreous AGE levels have mainly been measured in DR patients with proliferative diabetic retinopathy (PDR) or diabetic macular edema (DME) and have always been compared to non-diabetic controls undergoing vitrectomy for several eye conditions. Currently, it is unclear whether vitreous AGE levels are already elevated in diabetes before the development of PDR or DME.

The aim of the present study was to investigate several AGEs (pentosidine, CML, CEL and MG-H1) and α-dicarbonyls (3-DG, MGO, and GO) in the vitreous of type 2 diabetes patients (T2DM) without PDR in relation to non-diabetic controls. We examined vitreous samples obtained during vitrectomy from T2DM patients with a rhegmatogenous retinal detachment (RRD) and compared these to non-diabetic controls with comparable retinal detachment severity. Pentosidine is a cross-linking AGE which is thought to serve as an adequate marker for overall AGE levels[11,12]. In addition, DR could be influenced by AGEs through RAGE activation and, therefore, we also analysed several AGEs which have been shown to interact with RAGE[13,14].

## Materials and methods

### Study population

Participants were initially recruited in a prospective cohort study in which 410 RRD patients were included between November 2013 and August 2015 in our tertiary referral center (NTR4289). All patients gave written informed consent, and the study was approved by the Medical Ethics Committee of the University Medical Center Groningen and adhered to the Declaration of Helsinki. The present cross-sectional analysis is a sub-study hereof that addresses the secondary explicated research aim mentioned above. In this sub-study, 31 T2DM patients were 1:2 matched on age[15], estimated glomerular filtration rate (eGFR), smoking[16], intra-ocular lens implantation[17], and proliferative vitreoretinopathy (PVR)[17,18] to 62 non-diabetic patients. Diabetes mellitus was defined by criteria from the American Diabetes Association[19] or twice measured values of HbA1c ≥48 mmol/mol. Absence of diabetes and prediabetes in the non-diabetic patients was ascertained by absence of a history of diabetes, absence of anti-diabetic medication usage, and by presence of a normal HbA1c level (<42mmol/mol) and a normal non-fasting glucose level (≤ 7.8 mmol/l).

Clinical data and general characteristics were obtained by chart review and questionnaires. The following exclusion criteria applied to all subjects: known renal disease with impairment of renal function (eGFR <60 ml/min), dialysis treatment, history of renal transplantation[20], current infection or active inflammatory disease[21]. Presence of DR was established by fundus photography or by ophthalmic examination by an ophthalmologist. Both pre- and postoperatively, patients were examined accurately by fundoscopy. When DR was not specifically mentioned in latest notes of the ophthalmologist or retinal surgeon, it was assumed that DR could be classified as absent or minimal background DR.

### Standard laboratory assessments

Non-fasting venous blood was collected by venipuncture. Plasma glucose, HbA1c, serum creatinine and C-reactive protein (CRP) were measured using standard procedures. Renal function was evaluated by eGFR calculated by the *Modification of Diet in Renal Disease* (MDRD) formula: eGFR = 186 × [serum creatinine (μmol/L) × 0.0113]^−1.154^ × age^-0.203^ (× 0.742 for women). CRP was measured to ensure that no active infection or inflammatory disease was present.

### Vitreous samples and measurement of AGEs

At the start of the vitrectomy procedure, undiluted vitreous samples were collected from the midvitreous by aspirating the vitreous manually through a syringe connected to the vitrectome. The samples were frozen within 15–30 minutes and stored at -80°C until further use. Measurement methods of AGEs and *α-*dicarbonyls have been described in detail elsewhere: pentosidine was measured using high performance liquid chromatography (HPLC) with a fluorescence detector[22]; lysine and protein-bound and free CML, CEL, and MG-H1 were measured using ultra performance liquid chromatography tandem mass spectrometry (UPLC MS/MS)[23]; derivatized 3-DG, MGO, and GO were analyzed by UPLC MS/MS[24].

### Skin autofluorescense

Skin autofluorescence (SAF) was measured on the left forearm using the AGE Reader (DiagnOptics Technologies BV, Groningen, The Netherlands), a non-invasive desk-top device using the characteristic fluorescent properties of certain AGEs to estimate the level of AGE accumulation in the skin. Technical details concerning the optical technique have been extensively described elsewhere[25].

### Ophthalmic characteristics

Characteristics of retinal detachment were determined during surgery. Surface area of detached retina in relation to total retinal surface was scored in quartiles. Proliferative vitreoretinopathy (PVR) was graded A to C, according to the Retina Society PVR classification[26]. When PVR grade A was present, patients were classified as ‘non-PVR’; when PVR grades B or C were present, patients were classified as ‘PVR’.

### Statistical analysis

Sample size was based on previously reported pentosidine concentrations in diabetes and control patients[27] in which the effect size was 1.1. Based on α = 0.05 and power = 80%, the minimum number of subjects needed in this study was 25 T2DM patients and 50 control patients. Data obtained from each sample group were presented as mean and standard deviation (SD) or as median and interquartile range (IQR). Differences between T2DM patients and controls were tested using χ^2^ tests for categorical variables, t-tests for normally distributed continuous variables and Mann-Whitney U tests for remaining variables. Spearman correlation coefficients were used for a correlation analysis. Statistical significance was accepted at p < 0.05. Statistical analyses were performed using SPSS version 22 (IBM Corp., Armonk, NY, USA).

## Results

Clinical characteristics of the study population stratified for T2DM patients and controls are shown in Table 1. There was no significant difference in clinical characteristics between the two subgroups, except for body mass index, non-fasting glucose, and HbA1cFurthermore, there was no significant difference in intra-operatively present characteristics of retinal detachment (i.e. surface area of detachment, PVR grade and detachment duration), indicating comparable disease severity between the subgroups.

**Table 1:** Clinical characteristics of the study population.

Characteristic	T2DM (n = 31)	Control (n = 62)	*P* Value
Age, *y*	64 ± 7.6	63 ± 8.1	NA
Gender, male, *%*	74.2	79.0	0.599
**BMI, *kg/m***^***2***^	**27 ± 3.9**	**25 ± 3.4**	**< 0.001**
Duration of diabetes, *y*	2.2 (0.3–7.4)	-	NA
Systolic blood pressure, *mmHg*	131 (120–145)	135 (130–145)	0.463
Current smoker, *%*	12.9	11.3	NA
Intra ocular lens implantation, *%*	45.2	46.8	NA
Surface area of detachment >50%, *%*	45.2	50.0	0.764
PVR grade B & C, *%*	29.0	17.7	NA
Detachment duration, *days*	9 (7–18)	8 (5–16)	0.191
**Non fasting glucose, *mmol/L***	**7.1 (6.5–9.4)**	**5.9 (5.6–6.3)**	**< 0.001**
**HbA1c, *mmol/mol***	**50 (45–60)**	**37 (35–39)**	**< 0.001**
eGFR, *ml/min*	91 (75–106)	85 (75–99)	NA
Skin autofluorescence, *AU*	2.5 (2.2–3.0)	2.2 (2.1–2.6)	0.070

For categorical variables, data are displayed as percentages and p-values are based on χ^2^ tests. For normally distributed continuous variables, data are given as mean ± SD and p-values are based on t-tests. For remaining variables, data are shown as median (interquartile range) and p-values are based on Mann-Whitney U tests. Note: no statistical tests were performed on the parameters on which both groups were matched. T2DM, type 2 diabetes mellitus; NA, not applicable; BMI, body mass index; PVR, proliferative vitreoretinopathy; eGFR, estimated glomerular filtration rate.

### Characteristics of T2DM patients

Thirty-one T2DM patients (age range: 43–84 years) participated in the study, including 6 who were newly diagnosed with diabetes at inclusion. Median diabetes duration, as recorded by the general practitioner, was 2.2 (0.3–7.4) years. Maximum diabetes duration was 26.3 years. Two patients were treated with insulin only, 3 patients with a combination of insulin and metformin, 10 patients with metformin and 6 patients with a combination of 2 oral anti-diabetics. The remaining 10 patients were not treated medically or had only received lifestyle advice before inclusion. In the T2DM subgroup, 61% of the patients used anti-hypertensives and 48% of the patients used statins. Microvascular complications were present in 2 patients: 1 patient had diabetic neuropathy and 1 patient had non proliferative DR. In the remaining 30 patients, DR was classified as absent or minimal background retinopathy. Six patients had a history of myocardial infarction or cerebrovascular accident.

### Characteristics of controls

Sixty-two non-diabetic patients (age range: 44–83 years) formed the control subgroup. In this group, 27% of the patients used anti-hypertensives and 15% of the patients used statins. Furthermore, 4 patients had a history of myocardial infarction or cerebrovascular accident. Medication use of both anti-hypertensives (p = 0.002) and statins (p < 0.001), but not history of macrovascular events (p = 0.058), was significantly lower in the control group compared to T2DM.

### Vitreous AGEs and α-dicarbonyls

Biochemical characteristics of the vitreous stratified for T2DM patients and controls are shown in Table 2. Correlation analysis between AGEs and α-dicarbonyls on the one hand and general characteristics (like age, gender, HbA1c, and eGFR) on the other hand revealed some weak to moderate correlations. In the T2DM subgroup, 3-DG (r = 0.592, p = 0.001) was associated with HbA1c, while pentosidine (r = –0.022, p = 0.908) and the other AGEs and α-dicarbonyls were not. Additionally, SAF was not associated with any of the vitreous AGEs and α-dicarbonyls in the T2DM subgroup. Further results of the correlation analysis are not shown.

**Table 2:** Biochemical characteristics of the vitreous.

Characteristic	T2DM (n = 31)	Control (n = 62)	*P* Value
Lysine, mmol/L	0.56 (0.37–1.52)	0.66 (0.41–1.13)	0.993
Protein-bound AGEs			
**Pentosidine, *μmol/mol lysine***	**2.20 (1.59–3.20)**	**1.59 (1.18–2.52)**	**0.012**
CML, *μmol/mol lysine*	255 (199–298)	223 (179–318)	0.222
CEL, *μmol/mol lysine*	127 ± 66.3	122 ± 64.3	0.717
MG-H1, *μmol/mol lysine*	750 (452–1264)	734 (383–1383)	0.883
Free AGEs			
CML, *nmol/L*	78 (57–92)	77 (58–95)	0.839
CEL, *nmol/L*	71 ± 21.7	65 ± 21.1	0.198
MG-H1, *nmol/L*	145 (100–234)	181 (133–247)	0.156
α-dicarbonyls			
**3-DG, *nmol/L***	**809 (714–1043)**	**615 (531–764)**	**< 0.001**
MGO, *nmol/L*	231 (197–289)	234 (199–274)	0.961
GO, *nmol/L*	466 (330–747)	431 (246–675)	0.563

For normally distributed continuous variables, data are given as mean ± SD and p-values are based on t-tests. For other variables, data are shown as median (interquartile range) and p-values are based on Mann-Whitney U tests. T2DM, type 2 diabetes mellitus; CML, N^ε^-(carboxymethyl)lysine; CEL, N^ε^-(carboxyethyl)lysine; MG-H1, 5-hydro-5-methylimidazolone; 3-DG, 3-deoxyglucosone; MGO, methylglyoxal; GO, glyoxal.

Comparison of AGE levels between the subgroups showed significantly increased vitreous protein-bound pentosidine in T2DM patients (2.20 vs. 1.59 μmol/mol lysine, p = 0.012). Both protein-bound and free CML, CEL, and MG-H1 were not significantly different.

Comparison of α-dicarbonyl levels between the subgroups showed significantly increased vitreous 3-DG concentrations in T2DM patients (809 vs. 615 nmol/L, p < 0.001). Vitreous MGO and GO were not significantly different.

## Discussion

This study addressed the accumulation of several AGEs and their potential precursors (α-dicarbonyls) in a unique body compartment: the vitreous body. Our results show that both protein-bound pentosidine and 3-DG were significantly elevated in T2DM patients as compared to non-diabetic controls matched for age, eGFR, smoking, intra-ocular lens implantation and PVR. However, the levels of other protein-bound AGEs (CML, CEL, and MG-H1), free AGEs (CML, CEL, and MG-H1), and other α-dicarbonyls (MGO and GO) did not differ between the two groups.

It has been proposed that AGEs and α-dicarbonyls may be considered as major initiators of retinal microvascular complications in T2DM. Enhanced reactive oxygen species (ROS) generation induced by AGE-RAGE interaction appears to be a likely cause of pericyte loss, the earliest histopathological hallmark of DR. Furthermore, AGEs are considered to stimulate vascular endothelial growth factor (VEGF) expression in pericytes, which is considered to promote neovascularization in DR[28,29]. Moreover, it has been suggested that AGEs might contribute to persistent central vitreo-retinal adhesions[17], which leads to vitreoretinal traction that can exacerbate the course of DR.

Several reports have shown elevated levels of pentosidine (measured by HPLC or enzyme-linked immunosorbent assay (ELISA)) in the vitreous of patients with PDR compared to non-diabetic controls[27,30,31]. However, one study[32] reported that the significantly higher levels in the T2DM group disappeared when levels of pentosidine (measured by HPLC) were corrected for vitreous protein concentration. This is important because this may suggest that the increase in pentosidine concentration might simply reflect alterations in the blood-retinal barrier in DR. Furthermore, the aging vitreous consists of areas of synchisis and syneresis[33,34], which could lead to sampling errors while taking a vitreous biopsy.

In the current study, pentosidine concentrations expressed per mmol of lysine residues were significantly elevated in T2DM patients. Since lysine is used as a proxy for the total amount of protein available for AGE modification[35,36] expression of AGEs per mmol of lysine provides an adjustment for differences in protein amount in the vitreous biopsy. The current study is also of value because it shows that pentosidine levels are already elevated in T2DM patients without extensive microvascular damage to the retina.

Concerning 3-DG in diabetes, no previous reports have addressed its level in other tissues than plasma. In plasma, several studies have reported elevated levels of 3-DG in both T1DM and T2DM[37–39], and 3-DG has been suggested to play a role in the development of vascular complications[6,40]. In agreement with these previous reports, the current study shows elevated 3-DG levels in the vitreous of T2DM patients.

Skin autofluorescence, as an extensively validated marker of skin accumulation of AGEs, tended to be greater in T2DM patients compared to controls. This is in line with previous publications[41,42] in which skin autofluorescence was increased in patients with diabetes, particularly in case of complications. The difference in skin fluorescence in our study was just not significant, which is probably due to the short diabetes duration and limited numbers of patients and complications.

Our observation that other protein-bound AGEs (CML, CEL, and MG-H1) and α-dicarbonyls (MGO and GO) were not elevated in the vitreous of T2DM patients compared to controls was unexpected. Of these AGEs and α-dicarbonyls, only protein-bound CML has been previously investigated in the vitreous of patients with diabetes. In that study[10], CML was found to be increased in PDR patients compared to controls. In addition, increased levels of several protein-bound AGEs and α-dicarbonyls have been found in the plasma of patients with diabetes[43]. Furthermore, in most studies, accumulation of AGEs in skin and serum are strongly associated with the severity of diabetic complications[44].

Since free AGEs are breakdown products of protein-bound AGEs, the absence of elevated levels of free AGEs indicates that the lack of differences in protein-bound CML, CEL, and MG-H1 in the current study is not a result of differences in degradation of these products. To illustrate, when degradation of proteins in the vitreous would be present to a greater extent in T2DM patients, one would expect that free AGEs would be elevated in these patients.

Several factors should be considered in interpreting the noted dissimilarities of our results with previous research. First, our study group consisted of T2DM patients with a relatively short duration of diabetes and without PDR. Second, it is important to distinguish between the results of measurements in different body compartments. It is not correct to directly compare results of plasma values to vitreous values because of differences in tissue turnover. Moreover, the vitreous is unique in its supply of nutrients and exposure to plasma components because of the blood-retinal barrier. Third, methodological differences in AGE measurements may contribute to discrepancies in results, especially when AGE levels are not expressed per protein content of the samples. Further, limited blood contamination in the vitreous samples of 4 diabetes and 5 control patients could have influenced our results. However, previous research indicated that limited blood contamination did not confound AGE measurements in the vitreous of RRD patients. Additionally, several frequently used drugs (such as anti-hypertensives[45] and statins[46]) have been shown to inhibit AGE formation in vitro and in vivo[47]. Although the influence of these drugs on AGE accumulation in human tissues with slow turnover has to be further elucidated, the more frequent use of anti-hypertensives and statins in T2DM patients may have masked our results by lowering AGE levels in these patients. Finally, our current study size may be too small to detect differences in other AGEs than pentosidine, since our sample size calculation was based on previously reported pentosidine values only.

Overall, it is difficult to provide a concise explanation as to why most of the measured AGEs and α-dicarbonyls in the current study were similar in the vitreous of the two patient groups while only pentosidine and 3-DG were elevated in the diabetes group. Correlation analysis showed a moderate correlation of 3-DG with HbA1c in the diabetes subgroup, indicating a relation with glycaemic control. This observation suggests an influence of mechanisms outside the eye on vitreous AGE content. Whether this association is causal, or merely a reflection of the underlying disease process cannot be determined by the current study. The generally weak relations between AGE levels in tissues with slow turnover and plasma stress the need for differentiating the behaviour of different AGEs and α-dicarbonyls within and between different tissue compartments. Another recent illustration of this need for differentiating AGE behaviour is given in a study which showed a close correlation of SAF and plasma pentosidine, but not of plasma CML and CEL, with arterial pulse wave velocity[48].

Because of its cross-sectional design, the current study could not address the causal role of AGEs in DR. Prospective studies are needed to investigate the role of AGEs in the development of DR and their potential to influence the transition of retinopathy from the non-proliferative to the proliferative state.

The major strength of the current study is that several AGEs and α-dicarbonyls were measured with state-of-the-art techniques based on UPLC MS/MS. Furthermore, only patients with RRD (with and without T2DM) were investigated in this study. This is an advantage to investigate the influence of diabetes on AGE accumulation in the vitreous per se. Moreover, it is usually hard to find a homogeneous diabetes group without PDR and a homogeneous control group for which vitreous samples are available since vitrectomy procedures are only performed in limited patient groups. On the other hand, the inclusion of only RRD patients is a limitation of this study. Care must be taken in extrapolating the results to T2DM patients and controls without RRD since the blood-retinal barrier may be disrupted to varying degrees in RRD[49,50]. Furthermore, our results concerning free AGEs and α-dicarbonyls should be interpreted with caution. Since the exact behaviour of free AGEs and α-dicarbonyls in the vitreous is unknown, the results concerning these products could be subject to sampling errors.

In summary, this study shows that levels of protein-bound pentosidine and 3-DG were increased in the vitreous of T2DM patients with a relatively short duration of diabetes. Since AGEs have been suggested to be involved in the development of DR, this study provides a basis for future studies in DR by showing an overview of several specific AGEs and α-dicarbonyls in the vitreous of early diabetes and non-diabetic subjects. Prospective studies with standardized AGE measurements in diabetes patients are needed to further elucidate the exact role of different AGEs and α-dicarbonyls in the development of DR.
